# Clinical outcomes of low and intermediate risk differentiated thyroid cancer patients treated with 30mCi for ablation or without radioactive iodine therapy

**DOI:** 10.20945/2359-3997000000025

**Published:** 2018-03-23

**Authors:** Shirlei Kugler Aiçar Súss, Cleo Otaviano Mesa, Gisah Amaral de Carvalho, Fabíola Yukiko Miasaki, Carolina Perez Chaves, Dominique Cochat Fuser, Rossana Corbo, Denise Momesso, Daniel A. Bulzico, Hans Graf, Fernanda Vaisman

**Affiliations:** 1 Instituto Nacional do Câncer Instituto Nacional do Câncer Rio de Janeiro RJ Brasil Serviço de Endocrinologia, Instituto Nacional do Câncer (Inca), Rio de Janeiro, RJ, Brasil; 2 Universidade Federal do Paraná Universidade Federal do Paraná Hospital das Clínicas Curitiba PR Brasil Serviço de Endocrinologia, Hospital das Clínicas, Universidade Federal do Paraná (UFPR), Curitiba, PR, Brasil; 3 Instituto Nacional do Câncer Instituto Nacional do Câncer Rio de Janeiro RJ Brasil Serviço de Medicina Nuclear, Instituto Nacional do Câncer (Inca), Rio de Janeiro, RJ, Brasil

**Keywords:** Thyroid carcinoma, radioiodine ablation, low activity

## Abstract

**Objective:**

To retrospectively evaluate the outcomes of patients with low and intermediate risk thyroid carcinoma treated with total thyroidectomy (TT) and who did not undergo radioiodine remnant ablation (RRA) and to compare them to patients receiving low dose of iodine (30 mCi).

**Subjects and methods:**

A total of 189 differentiated thyroid cancer (DTC) patients treated with TT followed by 30mCi for RRA or not, followed in two referral centers in Brazil were analyzed.

**Results:**

From the 189 patients, 68.8% was ATA low-risk, 30.6% intermediate and 0.6% high risk. Eighty-seven patients underwent RRA and 102 did not. The RRA groups tended to be younger and had a higher frequency of extra-thyroidal extension (ETE). RRA did not have and impact on response to initial therapy neither in low (*p* = 0.24) nor in intermediate risk patients (*p* = 0.66). It also had no impact on final outcome and most patients had no evidence of disease (NED) at final follow-up. Recurrence/persistence of disease was found in 1.2% of RRA group and 2% in patients treated only with TT (*p* = 0.59).

**Conclusions:**

Our study shows that in low and intermediate-risk patients, RRA with 30 mCi seems to have no major advantage over patients who did not undergo RRA regarding response to initial therapy in each risk group and also in long term outcomes.

## INTRODUCTION

Over the last decades, the incidence of DTC has increased significantly, especially of tumors smaller than 2 cm (1–3). Despite this, most of these patients have an excellent prognosis and a long followup during their lifetime (4,5). Nevertheless, rarely small tumors can metastasize and have an increased recurrence risk (6–8).

Recently, it has been advocated an individualized approach of DTC based on risk stratification (8–11) and a more selective use of RRA (12–15). In this sense, treatment for low-risk tumors tends to be less aggressive than high-risk tumors (16,17). Indeed, the use of RRA in low risk patients remains controversial, because it has not been shown to be beneficial in their management after complete surgical resection in some recent studies (18). More recent guidelines recommend a more careful use of radioiodine (RRA) due to its adverse effects, particularly chronic sialoadenitis and the increased risk of development of a second primary neoplasm (19,20).

In this sense, several studies have already shown that RRA with low RRA dose (30 mCi) is as effective as higher activities with excellent correlated remission rates (21–23). Mujammami and cols. summarizes the results of six studies, in which patients responded equally well to low iodine activity for RRA, when compared to higher activities, and the risk category was not a significant predictor of remission (low, intermediate and some high-risk patients without metastases) (7). However, there are authors who advocate the selective use of iodine, showing that it is not necessary in certain circumstances. In the study by Molinaro and cols., 63.6% of non-ablated low and intermediate risk patients evolved with remission during the follow-up period, without additional therapy, and all of these patients remained in remission through the study (24). Also, Schvartz and cols. failed to prove any benefit of RRA in survival in patients with low-risk DTC after thyroidectomy, reinforcing the idea that they should not be over-treated (22). Durante and cols., showed almost identical clinical response rates for low and intermediate risk patients, between subgroups with and without RRA (4). Similarly, our group had also previously shown that low and intermediate risk patients not treated with RRA had a very low risk of recurrence (25,26).

The aim of this study is to retrospectively analyze the follow-up of patients with thyroid carcinoma who were treated with TT and who did not receive RRA and compare them to patients who received a low dose of iodine (30 mCi).

## SUBJECTS AND METHODS

We retrospectively reviewed the medical records of 189 patients with DTC > 18 years old treated with TT without RRA or with low dose RRA (30 mCi) between 1975 and 2015. We included 61 patients treated in Hospital das Clínicas da Universidade Federal do Paraná (HC-UFPR), Curitiba, Brazil and 128 patients treated in National Cancer Institute (Inca), Rio de Janeiro, Brazil. A minimum of 18 months of followup after initial therapy was required for entry into the study, unless one of the clinical outcomes was reached before that time point.

The initial thyroid surgery was total thyroidectomy (TT) in all patients. Therapeutic neck dissections were only performed for clinically apparent abnormal cervical lymphadenopathy, since in both institutions, it is not routinely performed prophylactic neck dissections for DTC. RRA was performed within two to six months after surgery in 87 patients and 102 patients did not receive RRA. RRA therapy was administrated after thyroid hormone withdrawal (TWH) for 30 days or with recombinant human TSH (TSHrh) administration in selected patients, and under a low iodine diet. Preablation serum TSH, thyroglobulin (Tg) level and thyroglobulin-antibodies (Tg-Ab) titer were obtained in all patients and whole-body scans (WBS) were performed five to ten days after RRA.

This study was approved by the local research Ethics Committees.

### Follow-up

Patients were followed every 6-8 months during the first year and at 6-12 months intervals thereafter at the discretion of the attending physician based on the risk of recurrence of the individual patient and the clinical course of the disease. Routine evaluation included serum TSH, serum Tg, Tg-Ab and neck ultrasound (US). For those patients with suspected local or distant metastasis, other imaging modalities such as computed tomography (CT) scan, or magnetic resonance imaging (MRI) and/or biopsies were performed as needed. Patients were treated and followed by the same group of physicians in each center.

### Laboratory studies

Serum Tg was measured postoperative and during follow-up in regular bases. We considered postoperative Tg, measurements performed at a minimum of 6-8 weeks after TT, since Tg usually reaches its nadir by 3 to 4 weeks postoperatively (9,15). Trend of non-stimulated Tg was evaluated at the same TSH levels and defined as: stable, decreasing, or increasing (> 20% over baseline).

At Inca, from 1977 to 1985, the functional sensitivity of the serum Tg assays was approximately 5 ng/mL. Between 1986 and 1997, a variety of Tg assays was used with functional sensitivities of approximately 1 ng/mL. From 1998 to 2001, a Tg assay with a functional sensitivity of 0.5 ng/L was employed. Starting in 2001 until 2010, serum Tg was quantified by a immunometric assay (Immulite) with a functional sensitivity of 0.2 ng/mL, and from 2010 until today, the functional sensitivity dropped to 0.1 ng/mL.

At HC-UFPR, from 1990 to 2004, serum Tg was quantified by a radioimmunoassay assay with a functional sensitivity of 0.9 ng/mL. From 2004 until today, serum Tg was quantified by a chemiluminescence assay with a functional sensitivity of 0.1 ng/mL.

### Risk stratification

Patients were stratified using the modified ATA 2015 risk stratification system (low or intermediate risk) (15).

Dynamic risk stratification was performed using the response to therapy assessment during the first 2 years of follow-up previous published by Tuttle and cols. and in the latest ATA guidelines (15,23). In patients treated with RRA, response to therapy were defined as: excellent response to therapy (negative imaging and suppressed Tg < 0.2 ng/mL and stimulated Tg < 1.0 ng/mL); indeterminate response (nonspecific findings on imaging studies, non-stimulated Tg detectable but < 1 ng/mL, stimulated Tg detectable but < 10 ng/mL); biochemical incomplete response (negative imaging and non-stimulated Tg > 1 ng/mL or stimulated Tg > 10 ng/mL); or structural incomplete response to therapy (structural or functional evidence of disease, with any Tg level) (9,15). For patients treated with TT without RRA, response to therapy definitions were: excellent response to therapy (undetectable Tg-Ab, negative imaging and non-stimulated Tg < 0.2 ng/mL or stimulated Tg < 2.0 ng/mL); indeterminate response (stable or declining Tg-Ab and/or nonspecific imaging findings and non-stimulated Tg detectable but 0.2-5 ng/mL, stimulated Tg detectable but 2-10 ng/mL); biochemical incomplete response (negative imaging and/or increasing Tg with similar TSH levels and/or increasing Tg-Ab and non-stimulated Tg > 5 ng/mL or stimulated Tg > 10 ng/mL); or structural incomplete response to therapy (structural or functional evidence of disease, with any Tg level) (9,11). Tg analysis were considered as following: Post operative undetectable Tg when Tg was below functional sensitivity of the Tg assay used at the time of surgery and to determinate the trend overtime we considered increase if either suppressed or stimulated Tg were rising, decline if both were declining or if one was stable and the other was falling and stable if both were stable. In those patients who did not have stimulated Tg repeated overtime, only suppressed Tg was considered. All of the patients had post operative stimulated Tg measured.

### Clinical outcomes

Clinical outcomes were defined as:

- No evidence of disease (NED): the absence of suspected images and Tg levels used to classify as excellent response to therapy.
- Recurrent/persistent SD, defined as: positive cytology/histology, highly suspicious lymph-nodes or thyroid bed nodules on the US (hyper-vascularity, cystic areas, heterogeneous content, rounded shape and enlargement on follow-up), or cross-sectional imaging highly suspicious for metastatic disease.
- Disease specific mortality: death related to the tumor or its treatment.

Additional outcomes evaluated were: need for additional therapy during follow-up (additional surgery and/or RRA), clinical outcomes after additional therapy and the trend of non-stimulated Tg after initial therapy without RRA.

### Statistical analysis

Continuous data is presented as mean and standard deviations with median values. For comparing medians nonparametric Mann-Whitney test was used and for categories we used Chi2 to compare 2 or multiple groups and Fisher's exact tests. Analysis was performed using SPSS software (Version 20.0 for MAC; SPSS, Inc., Chicago IL).

## RESULTS

The demographics, clinical features, risk stratification, initial management and clinical outcomes of the 189 patients included in the cohort are presented in Table 1. Considering the entire cohort, TT was performed as initial therapy in all patients, papillary thyroid cancer (PTC) was the most common histology and the majority of patients were female. Fifty-four percent (n = 102) of patients did not receive RRA and 46% (n = 87) did undergo low dose RRA (30 mCi). Patients treated with RRA were younger (49, range 18-86 years in the group without RRA vs 43, range 19-80 years for the RRA group) and microscopic extra-thyroidal extension (ETE) was more frequently observed (29.9% for RRA vs 14.7% without RRA) (*p* = 0.04 and *p* = 0.01, respectively). The groups did not differ in tumor size, presence of vascular invasion, lymphnode metastases (N1), tumor multifocality or ATA risk stratification. There was no statistical difference in median postoperative non-stimulated Tg and presence of Tg-Ab between the groups. However, presence of undetectable Tg postoperative during follow-up was significantly different (*p* < 0.001), observed in 65,7% of the patients treated without RRA and 49,4% of patients treated with low dose RRA. The Tg trend overtime decline in 67,6% of patients treated without RRA and 56,3% patients treated with low dose RRA (*p* = 0.13).

**Table 1:** Description of the cohort (n = 189)

		Without RRA (n = 102)	Low dose RRA (30mCi) (n = 87)	p-value
Age		49 (18-86)	43 (19-80)	**0.04**
Gender-female		93.1% (n = 94)	86.2% (n = 75)	0.09
Histology Papillary thyroid cancer		93.1% (n = 94)	95.4% (n = 83)	0.36
ETE		14.7% (n = 15)	29.9% (n = 26)	**0.01**
Multifocality		34.3% (n = 35)	39.1% (n = 34)	0.54
Size (cm)		1 (0.9-9)	1 (0.3-4.0)	0.21
Vascular invasion		8.8% (n = 9)	13.8% (n = 12)	0.35
N1		15.7% (n = 16)	23% (n = 20)	0.26
Post-operative non-stimulated Tg		1.25 (< 0.1-34)	0.77 (< 0.1-15)	0.59
Undetectable post-operative Suppressed Tg		65.7% (n = 67)	49.4% (n = 43)	**< 0.001**
Positive Anti-Tg		6.9% (n = 7)	8.0% (n = 7)	0.78
ATA 2016 risk stratification				
	Low	78.4% (n = 80)	57.5% (n = 50)	**0.04**
	Intermediate	20.5% (n = 21)	42.5% (n = 37)	
	High	1% (n = 1)	0	
Median follow-up (months)		40.5 (1-488)	49.6 (4-321)	0.63
Recurrence/persistence structural disease		1% (n = 1)	1.1% (n = 1)	0.55
Additional therapy		1% (n = 1)	2.3% (n = 2)	0.59
Response to therapy – first 2 years of follow-up				
	Excellent	68.6% (n = 70)	81.6% (n = 71)	0.08
	Indeterminate	26.5% (n = 27)	13.8% (n = 12)	
	Biochemical incomplete	2.9% (n = 3)	2.3% (n = 2)	
	Structural incomplete	2% (n = 2)	2.3% (n = 2)	
Tg trend over time (suppressed and/or stimulated)			
Decline		67.6% (n = 69)	56.3% (n = 49)	0.13
Clinical status at final follow-up				
NED without additional therapy		98% (n = 100)	98,8% (n = 86)	0.59
NED after additional therapy		1% (n = 1)	1.2% (n = 1)	
Recurrent/persistent of disease after additional therapy		0%	0%	
Recurrent/persistent of disease without additional therapy		1% (n = 1)	0%	
Death from disease		0%	0%	

Data are presented percentage (number) or median (range). ETE: microscopic extra thyroid extension; N1: lymph node metastases; Tg: thyroglobulin; Anti-Tg: anti-Tg antibody; ATA: American Thyroid Association; NED: no evidence of disease.

During follow-up, recurrence/persistence of disease was similar between groups, with recurrence/persistence in 1% of patients not treated with RRA and 1.1% in patients treated with low dose RRA (30mCi) (*p* = 0.55). The median follow-up was 40.5 and 49.6 months respectively (*p* = 0.63) (Table 1). In both groups, the majority of patients had an excellent response to initial therapy followed by indeterminate and only few patients had incomplete response, either biochemical or structural. At final follow-up the majority of patients had no evidence of biochemical or structural disease without additional therapy. There were no cases of disease-related deaths.

Regarding response to therapy in patients classified as low risk by the ATA risk stratification system (*n* = 130), excellent response was found in 94 patients and indeterminate response in 30 patients, as presented in the univariate analysis shown in Table 1. Papillary was the most common histology and the majority was female in both groups. Similarly, age and size of tumor were not statistically different among them. The median postoperative non-stimulated Tg was significantly statistically different (0.1 ng/mL in patients with excellent response, ranging from < 0.1 to 3.4 ng/mL *vs* 1.0 ng/mL in patients with indeterminate response, ranging from < 0.1 to 3.0 ng/mL, *p* < 0.001). The presence of lymph node metastasis at diagnosis was significantly different (7.4% in excellent response *vs* 13.3% in indeterminate response *vs* 66.7% and structural incomplete response, *p* = 0.04). When patients were analyzed according to their initial risk of recurrence (Tables 2 and 3), RRA treatment did not differ among patients with excellent and indeterminate response to initial therapy. Postoperative nonstimulated Tg was the only predictor of response to therapy in low and intermediate risk groups (Tables 2 and 3). Extrathyroidal extension, present only in the intermediate risk group, did not have impact on response to initial therapy even being more frequent in the group that underwent RRA.

**Table 2:** Response to therapy in low risk patients (n = 130) during the first 2 years of follow-up

LOW RISK Patients	Excellent (n = 94)	Indeterminate (n = 30)	Biochemical incomplete (n = 3)	Structural incomplete (n = 3)	p-value
Age (years)	44.5 (20-86)	45.5 (26-76)	42 (32-57)	53 (39-79)	0.61
Gender-female	89.4% (n = 84)	90% (n = 27)	100% (n = 3)	100% (n = 3)	0.72
Histology					
Papillary thyroid cancer	91.5% (n = 86)	94.3% (n = 33)	100% (n = 3)	100% (n = 3)	0.88
Post operative non stimulated Tg	0.1 (< 0.1-3.4)	1.0 (< 0.1-3.0)	N/A	N/A	**< 0.001**
Size (cm)	2.0 (0.1-9.0)	1.0 (0.2-6)	1.7 (x-2.0)	1.2 (1.1-1.4)	0.78
N1	7.4% (n = 7)	13.3% (n = 4)	0%	66.7% (n = 2)	**0.04**
RRA	43.6% (n = 41)	26.7%% (n = 8)	0%	33.3% (n = 1)	0.24

Data are presented as percentage (number) or median (range). Tg: thyroglobulin, N1: lymph node metastases, RRA: radioiodine, N/A: non available.

**Table 3:** Response to therapy in intermediate risk patients (n = 58) during the first 2 years of follow-up

Intermediate risk patients	Excellent (n = 46)	Indeterminate (n = 9)	Biochemical incomplete (n = 2)	Structural incomplete (n = 1)	p-value
Age (years)	42 (18-69)	36 (34-43)	34.5 (19-50)	59	0.16
Gender-female	91.9% (42)	66.6% (n = 6)	100% (n = 2)	100% (n = 1)	0.48
Histology					
Papillary thyroid cancer	97.8% (n = 45)	100% (n = 9)	100% (n = 2)	100% (n = 1)	0.96
Post operative non stimulated Tg	< 0.1 (< 0.1-0.5)	0.1 (< 0.1-0.2)	1.4	3	**< 0.01**
ETE	69.6% (n = 32)	55.5% (n = 5)	100% (n = 2)	100% (n = 1)	**0.89**
Size (cm)	2.1 (1.0-4.7)	2.3 (2.1-3.0)	1.5 (0.6-2.4)	1.4	0.92
N1	37% (n = 17)	22.2% (n = 2)	50% (n = 1)	0%	0.80
RRA	65.2% (n = 30)	44.4% (n = 4)	50% (n = 1)	100% (n = 1)	0.66

Data are presented as percentage (number) or median (range). Tg: thyroglobulin; N1: lymph node metastases; RRA: radioiodine.

Only 3 patients with low risk tumors had a biochemical incomplete response and 3 had structural incomplete within the first 2 years of follow-up. They are shown in Table 4. Also, 3 patients from the intermediate risk group had incomplete responses.

**Table 4:** Clinical characteristics of differentiated thyroid cancer patients with incomplete response to therapy within the first 2 years of follow-up (biochemical or structural)

	Pt1	Pt2	Pt3	Pt4	Pt5	Pt6	Pt7	Pt8	Pt9
Age (years)	79	53	39	57	42	32	59	50	19
Gender	F	F	F	F	F	F	F	F	F
Histology	PTC	PTC	PTC	PTC	PTC	PTC	PTC	PTC	PTC
Size (cm)	1.1	1.2	1.4	1.7	2.0	x	1,4	2,4	0,6
pN1	Yes	Yes	No	No	No	No	No	No	Yes
ATA risk	Low	Low	Low	Low	Low	Low	Intermediate	Intermediate	Intermediate
Radioiodine	No	Yes	No	Yes	No	No	Yes	No	Yes
Response to therapy	SI	SI	SI	BI	BI	BI	SI	BI	BI
Additional Therapy	No	Yes	Yes	No	No	No	No	No	Yes
Time to additional Therapy	x	18	120	x	x	x	x	x	34
Tg trend overtime	Decline	Decline	Decline	Decline	Decline	Decline	Stable	Stable	Increase
Follow-up (months)	20	18	384	16	28	139	12	23	39
Status at final follow-up	PD	NED-AT	NED-AT	NED	NED	NED	NED	NED	NED - AT

F: female; SI: structural incomplete; BI: biochemical incomplete; PD: persistent of disease; NED-AT: no evidence of disease-additional therapy; NED: no evidence of disease.

Stands out that the only patient with persistent disease (incomplete structural response) still survives with structural disease because it is a heterogeneous lymph node in the left anterior cervical region, near the sternal, which is suspect because of its characteristics (irregular margins) and size (1.4 x 1.2 x 1.3 cm), remains unchanged for more than one year; as well as without elevation of Tg.

## DISCUSSION

This study showed that in properly selected low and intermediate risk DTC patients, low activities of RRA did not have a significant impact on response to therapy and also final status of being disease free. Furthermore, in response to initial therapy this difference was also not seen when patients were analyzed according to their initial risk of recurrence separately. Previous studies, Hilo and ESTIMABL, have shown in randomized trials that low activities such as 30mCi are equally effective for low and intermediate risk patients as RRA (12,14). Vaisman and cols., thus, showed that the recurrence rate of properly selected low and intermediate risk DTC patients are very similar in patients who underwent RRA and those followed with no additional therapy after surgery (26). Furthermore, the study by Schvartz and cols., with a 10.3 years follow-up, showed no benefit of RRA after surgery on survival in a large cohort of patients with low-risk DTC (22). In the study by Durante and cols., the rates of complete clinical response were almost identical in the subgroup that received ablation and in the subgroup that did not receive (92.2% and 98.3%, respectively), reinforcing the low impact of RRA in response to therapy (4).

In the present study, patients treated with RRA were younger, and microscopic ETE was observed more frequently (*p* = 0.04 and *p* = 0.01, respectively). It is known that there is a tendency for an increase in the use of RRA in younger patients; however, most studies fail to show that isolated extra-thyroidal microscopic invasion increases recurrence significantly as an independent factor. Furthermore, the most recent recommendation is to avoid RRA in most patients when this is the only reason to treat with RRA (6). Moreover, in this present study, ETE did not have impact in response to initial therapy in the intermediate risk group, probably being only a factor that have influenced the decision of performing RRA, specially in the past.

In recent years, postoperative evaluation is becoming more important in decision making of RRA (13). This present study showed that the presence of undetectable non-stimulated Tg during follow-up was significantly different and more frequent in the non RRA group (*p* < 0.001), suggesting that independently of initial risk stratification, when postoperative Tg was negative, physicians were confortable to only follow these patients without RRA. Corroborating such findings, Webb and cols. concluded that low pre-ablation Tg should be considered a favorable risk factor in patients with DTC, suggesting that it may perhaps be used to select patients not candidates for RRA (15,27). In the same sense, Momesso and cols. (11) demonstrated that the value of non-stimulated Tg was the greatest predictor of recurrence/persistence of structural disease, which supports previous findings of patients not treated with RRA (10), in whom the decline or stability of non-stimulated Tg presented a good prognosis (18,26,28), being the best response in the first two years of follow-up, comparable to that at any time in the total follow-up of these patients (11). In the same study, there was no disease-specific mortality and low rates of recurrence/persistence of structural disease (11), as also occurred in the on-screen study, in which the majority of patients, more than 97%, had no evidence of disease (NED) at the end of the follow-up without additional therapy and without cases of death by the disease.

Over time, the trend of Tg values decreased by more than 55% in both groups. At the final follow-up, most patients had no evidence of biochemical or structural disease without additional therapy. In the study by Mujammami and cols., levels of pre-dose ablative Tg and biochemical response to primary therapy predicted favorable outcomes (7), with the majority of patients with elevated Tg levels and incomplete or indeterminate responses progressing to undetectable levels of Tg without further treatment (7). This has already been observed by Vaisman and cols. (10), who concluded that patients with incomplete biochemical response may be reclassified as without evidence of disease, without additional therapy beyond suppression of TSH with LT4. Similarly, Momesso and cols. showed that even patients with indeterminate response to therapy had a good prognosis, with no evidence of structural disease during follow-up, and could be observed conservatively (4,9,11,29). Current second generation Tg assays (0.05-0.1 ng/mL) have shown that patients with lowrisk DTC with undetectable baseline Tg rarely recur (5). In this sense, study published in 2016 by Janovsky (1) concluded that an excellent response to treatment can be confirmed by the trend of Tg and US, these being the best follow-up approaches, showing that the use of RRA only to achieve negative levels of Tg for surveillance is not necessary because it is not an isolated value, but the tendency of Tg during follow-up, the determining factor for both (1). Therefore, based on the above, it should be emphasized that the trend of Tg is highly suggestive of being a predictor of disease-free follow-up.

When analyzing the low-risk patients separately, patients with excellent response had lower values of postoperative Tg than patients with indeterminate response. Webb and cols. (27) examined the predictive value of a single dose of serum Tg immediately prior to RRA as a subsequent disease free state and concluded that this evaluation is an inexpensive tool with a high negative predictive value which could be used to select patients not RRA candidates (15,27).

The presence of lymph node metastasis at the time of diagnosis was more frequent in those patients with indeterminate response to initial therapy and less frequently in those with excellent response, demonstrating that such presence may negatively interfere in the response to treatment. However, based on current ATA recommendations (15) there is no indication for RRA in low risk patients with up to 5 micro-metastases to lymph nodes. Furthermore, Wang and cols. demonstrated that properly selected patients, even with affected lymph nodes, should not necessarily receive RRA (30,31).

It should be noted that the present work has some limitations. As it is a retrospective and non-randomized trial, selection bias based on the assistant physician to perform RRA could occur. However, the basic characteristics are similar in both groups. Furthermore, there were changes in ultrasound technology and Tg assays as mentioned before that could interfere in initial response to therapy classification, however, the most clinically relevant endpoints, which are recurrence, and structural incomplete response is less influenced by Tg values. Other important limitation is the follow – up period. As known, the majority of the recurrences occur with the first 2-5 years, however, some of those can occur later on. Further prospective studies and with longer follow-up periods are needed to validate our findings. In addition, most of the studies with 30mCi (12,14) evaluate the efficacy of ablation not correlating this with recurrence rates. In our study, we correlate low activities with response to therapy, which is a parameter with well establish correlation with long term prognosis (8,9,10,15).

In conclusion, the present study shows that in low and intermediate risk patients, the treatment with 30mCi of radioiodine for ablation might appear to make a difference in response to therapy, however, when longer follow-up is analyzed, RRA activities at ablation seems to have no advantage over patients who did not undergo RRA with long term outcomes. As a secondary endpoint, in these patients an excellent response to treatment may be assured by the postoperative nonstimulated Tg values analyzed as predictors of diseasefree follow-up.
